# Spinal Endermic Medication

**Published:** 1837

**Authors:** G. J. Cowles

**Affiliations:** Florence, Ala.


### Art. V.—Spinal Endermic Medication.

*Cases illustrating the efficacy of medicinal applications over the region of the Spine.* By Dr. G. J. Cowles, of Florence, Ala.

(Continued from the last number.)

*Case xiv.*—In the month of March, 1835, I was called to visit Mr. ——, a middle aged man, of regular habits, who generally enjoyed good health. A few days before, he was suddenly attacked with the following symptoms:—A chill, followed by fever, which subsided in 12 or 15 hours, with perspiration; but a violent pain ensued in the right hip, and speedily affected the whole limb: which was soon perceived to be hot, swollen, tender to the touch, and incapable of voluntary motion; no pitting followed pressure with the finger, and there was no redness except about the inner ankle; he was worse at night, and extremely restless till near morning; his pulse beat 100 in a minute, quick and rather strong; his tongue was white and moist; thirst considerable; appetite lost, with occasional nausea; bowels costive; urine turbid and offensive; the abdomen, especially in the course of the spermatic cord, tender on pressure; finally, there was a daily exacerbation of all the symptoms. The attack appeared to have been produced by exposure to inclement weather. The course of the spine, especially its lumber and sacral portions, was found to be tender on pressure, and under the application of a sponge dipped in hot water. Its heat was above the natural degree. The spinal pressure excited great pain in the affected limb. Bleeding by cups over the spine, for three successive days, followed by a blister, and a poultice, were prescribed, with saline aperients and a free use of barley water; at the same time, the limb was kept besmeared with olive oil. His recovery was rapid.

*Case xv.*—I was called to see a lady in 1836, who had suffered uninterrupted ill health since the birth of a child, five months before. She had a fever of an intermitting type, with wandering pains in the limbs, numbness of the thighs, coldness of the feet, burning in the hands, coated tongue, costive bowels and gastric derangement. Little or nothing being done to remove these symptoms, she was at length attacked with a violent chill, succeeded by high fever, with acute pain in the back, shoulder and arm, sometimes darting into the chest. The affected arm, and occasionally the side of the chest, exquisitely sensitive to pressure, so that even the weight of the bed clothes was insupportable to her. The arm swelled, but exhibited no redness, except a spot near the shoulder; it lost all power of motion; its heat was above that of other parts of the body. At this time her fever abated only towards morning, when some perspiration came on. Her pulse was considerably over 100, in a minute, quick and resisting; her tongue was covered with a light brown coat; her stomach was irritable, with occasional nausea and vomiting; she had flatulence, heartburn and costiveness; there was a feeling of tightness in the chest, and a fixed pain in the occiput, extending occasionally to the eye and other parts of the face and head. The secretion of milk had nearly ceased. With all these symptoms, she was little altered in appearance, but excessively nervous.

The illness of this lady was by herself traced up to her last labor, which was exceedingly protracted, and she continued after it to feel exhausted, nervous and feverish. On examining the spine, I found the skin over the upper dorsal and lower cervical vertebræ reddened, slightly thickened or raised, and very tender on pressure, which greatly increased the pain in her arm and chest, excited coughing and nausea, and depressed the pulse. No other portion of the spine appeared to be tender, but there was alternate heat and coldness of the feet and legs, which seemed to indicate the inferior spinal nerves were affected at their origins. *Treatment.*—Three large cups, with previous scarification, were applied over the spine, and repeated several times, at intervals of 12 hours, the parts being kept covered with warm emollient poultices; warm olive oil was applied to the affected side and arm, and at length counter irritants over the spine. The internal remedies were tartar emetic, calomel and other aperients and opium. Her recovery was rapid; but she remained for some time subject to slight pectoral symptoms, which were relieved by friction. This case seems to have had some of the characters of *phlegmasia dolens.*

*Case xvi.*—Mr. H., of delicate constitution, but generally healthy, in 1834, had febrile symptoms, with abdominal and pelvic derangements; which, under mild treatment, measurably subsided, but good health was by no means restored. When called to see him, subsequently, I found that he labored under fixed but not violent pain in the loins, numbness in the thighs, great sense of weakness, with pain in his legs, on motion; general exhaustion under exercise, with palpitation of the heart; throbbing in the abdomen; which was contracted and tender, especially in the region of the spermatic cord, the bladder and the perinœum; on examination, the prostate gland appeared to be swollen and painful, and he was found to have hœmorrhoidal tumors; the urine turbid and offensive, with occasional mucous discharges from the urethra; a coldness of his feet was often succeeded by the sensation of their being brought into contact with a hot body; there was, also, burning in the hands, and wandering pains sometimes spread through the head and shoulders; his tongue was pale, and partly covered with a brown fur; small ulcers in the face appeared, disappeared and returned, spontaneously; his thirst was considerable, appetite craving, digestion tolerable; bowels too open; emaciation great; countenance palid; he had frequent, profuse acid sweats; his pulse was frequent, sudden and rather hard; generally he was exceedingly irritable and impatient. These symptoms resisted the prescriptions of his medical attendants, and I was myself about to abandon the case as hopeless, when an accident directed my attention to his spine. He had, to use his own language, lain so long on his back, as to "ride it sore from one end to the other." An examination was immediately made, and many of the vertebræ, particularly the sacral and upper dorsal, were found to be tender; there was, also, a considerable degree of lateral curvature of the spine. Counter irritation over all the tender vertebræ was immediately commenced and continued for several weeks; blisters and poultices were applied to the perinœum; and stimulating liniments rubbed over the abdomen. Flax-seed tea and other mucilaginous drinks were used in connexion with alterants addressed to the abdominal organs; and, finally, tonics, a generous diet and gentle exercise in the open air were directed. This course soon relieved him from all alarming apprehensions, and in a few months he was entirely restored, and has enjoyed good health ever since.

*Case xvii.*—The following case has many points in common with the last. The patient was a young laboring man, to whom I was called in April 1836. He had been much exposed in various ways, and suffered undefinable bad health, when about the time I was called in, he was awakened one night with a chill, followed by fever, pain in the back, limbs, abdomen, and head, with thirst, dysuria, hæmaturia, a certain degree of blenorrhœa and great agony in the pelvis, the pains passing to the thighs; cold feet and hot hands; abdomen drawn up and tender in the hypogastric and iliac regions; the pulse was from 85 to 90 and quick, but not full; the force of the heart constantly too great; there was weakness of vision, with occasional pain in the eye-balls; the muscles of one shoulder were enfeebled or slightly paralyzed, so that it hung lower than the other. This young man had been induced to believe that he labored under stone in the bladder, but from the concourse of symptoms I presumed on the existence of spinal irritation. Examination demonstrated this, as under pressure, friction and the application of the hot sponge, the lumbar and sacral vertebræ, were found to be tender, and the experiments excited pain, not only in the spine, but in the distal extremities of the nerves; the same applications to the dorsal and cervical vertebræ, also, produced pain in the arms, head and chest, although there was no tenderness in those portions of the spine; I should have stated that he exhibited a sort of protrusion of the eye-balls, which were suffused with blood, while his face was pale and emaciated; there was likewise much digestive derangement, and he labored under hæmorrhoidal disorders; in short, his situation was every way deplorable. Blisters were applied over the lower part of the spine, followed by poultices, with rubefacients to the higher parts; low diet was directed, with mucilaginous drinks, and perfect rest; but he took very little medicine. He soon began to recover, and was entirely restored.

*Case xviii.*—Within the present year a young lady became my patient, with the following symptoms. For months she had labored under what was called dyspepsia, with wandering pains in the chest and limbs, she was debilitated and nervous, palid and emaciated, had palpitation of the heart, drowsiness, irritable temper, amenorrhœa, leucorrhœa, burning sensation of the hands and feet, fixed pain in the top of the head, eyes, jaw and face subject to pains, and one side of the last slightly paralyzed—also, slight paralysis in the muscles of the shoulder. Medicines had been resorted to in this case with some benefit, but blisters and other revulsants to the *seats* of pain, produced but temporary relief, and sometimes only translated it to another part. Applications had also been made to the spine; but from some cause or other, not to the tender portions, and with an aggravation of the disease. On examination, I found two curvatures of the spine, but no *great* soreness or pressure; the experiment, however, excited cough, smarting in the eye, depression of the action of the heart, and left her decidedly worse for 24 hours. The spinal treatment was employed with manifest benefit; the blue mass and aloes were also administered; and, in her convalescence, sub carbonate of iron, a generous diet, and free exercise in the open air.

In a subsequent number of the Journal I propose to narrate some other cases, falling into the same class with the foregoing. I cannot say whether diseases of spinal origin, or with spinal complication, are more frequent in Florence and its vicinity than other parts of the country, but I have certainly found them of very frequent occurrence. Nor can I affirm that all of those which I have detailed, are from original spinal derangement; for it cannot be denied, that the spine is very likely to become involved from disease in the organic extremities of the nerves. Even in this instance, however, it is quite possible that applications made near the spinal extremities of the nerves may be more beneficial than if made to any other part.—At all events, I cannot be mistaken in the practical *fact*, of having derived very great advantage from applications over the spinal column. The local detraction of blood has been the chief of these; but I have found the subsequent application of warm emollient poultices to add greatly to the efficacy of the first application.

In conclusion, I wish such of the readers of the Western Journal as may think the cases I have reported worthy of their attention, to refer to case No. III. p. 194, in the last number, and add to the treatment the omission, that before cupping the patient was bled three times.

November 1837.

